# Bioinformatics Goes to School—New Avenues for Teaching Contemporary Biology

**DOI:** 10.1371/journal.pcbi.1003089

**Published:** 2013-06-13

**Authors:** Louisa Wood, Philipp Gebhardt

**Affiliations:** 1 Training Team, EMBL-European Bioinformatics Institute, Wellcome Trust Genome Campus, Hinxton, United Kingdom; 2 European Learning Laboratory for the Life Sciences, European Molecular Biology Laboratory, Heidelberg, Germany; Whitehead Institute, United States of America

## Abstract

Since 2010, the European Molecular Biology Laboratory's (EMBL) Heidelberg laboratory and the European Bioinformatics Institute (EMBL-EBI) have jointly run bioinformatics training courses developed specifically for secondary school science teachers within Europe and EMBL member states. These courses focus on introducing bioinformatics, databases, and data-intensive biology, allowing participants to explore resources and providing classroom-ready materials to support them in sharing this new knowledge with their students.

In this article, we chart our progress made in creating and running three bioinformatics training courses, including how the course resources are received by participants and how these, and bioinformatics in general, are subsequently used in the classroom. We assess the strengths and challenges of our approach, and share what we have learned through our interactions with European science teachers.

## Introduction

In life sciences today, access to biological data is a standard part of research due to the development of high-throughput techniques generating vast amounts of data. This has revolutionised biology and meant a step change for how biological research is performed and data recorded and shared. Recent years have witnessed a rapid growth of databases holding data on all aspects of biology and the development of a range of tools and services to allow users to make sense of the data [1]. So far, these resources have largely remained the specialist domain of researchers. Training in using bioinformatics resources is provided for researchers, and coverage of bioinformatics is increasing at the undergraduate level [2]–[4]. However, we believe that access to biological data also offers opportunities in secondary science education by introducing students and their teachers to bioinformatics, authentic scientific inquiry, and original data [5]. Although generally aware of bioinformatics and the large quantities of biological data that are generated by new research technologies, teachers are still uncertain about how to connect these developments with the science they teach in the classroom. Bioinformatics and large-scale biological data are also largely absent from biology curricula.

Exciting hands-on encounters with state-of-the-art molecular biology techniques and interactions with active research scientists help bridge the gap between research and schools. Being in the unique position to connect scientific expertise and research findings directly with educational outreach activities, EMBL's European Learning Laboratory for the Life Sciences (ELLS) offers multifaceted training opportunities, including the LearningLAB training courses for teachers, webinars, the EMBL Insight Lectures series, the EMBLog teacher portal, and a repository for teaching resources. All of these aim to provide secondary school science teachers with the practical expertise and theoretical knowledge of how to bring concepts of molecular biology into the classroom.

ELLS's rationale for targeting teachers is based on their role as multipliers to students over several year groups and also within professional teaching networks. Directly engaging with teachers shortens the time it takes to bring new scientific findings to the classroom. Teachers can find it challenging to keep up with the pace of contemporary science developments, often lack direct links to centres performing basic research, and can feel isolated in their professional practice [6]. Providing a direct connection to current research and the researchers behind it is a core feature of our courses [7]. The teachers act as knowledge transformers, taking information from the expert source, “the horse's mouth,” and delivering it to their students as living science. In this way, the teachers can catalyse an increased interest in science among young people and thereby help inspire the next generation of scientists.

In order to raise teachers' awareness of bioinformatics and new ways to visualise and explore biological concepts, ELLS and EMBL-EBI have jointly run a series of teacher training workshops since 2010 (please see Text S1 for background information about EMBL, ELLS, and EMBL-EBI). The target audience for our courses is predominantly European secondary school science teachers. The European focus of EMBL creates a fruitful environment for the exchange of experiences between teachers from different countries. In order to be able to meaningfully connect bioinformatics with their practical teaching, we target teachers of older secondary school students, ideally 16 years and above, as it is at this stage when essential concepts such as the DNA code, replication, proteins, and other biological molecules are covered in detail as part of the curriculum. We also accept applications from individuals who are not teachers but who are involved in providing educational outreach to teachers and school groups as another way of disseminating the course knowledge more widely. For more details on how we select the course participants, please see the section “How we design and run bioinformatics courses for teachers” in Text S1.

An essential step during the process of bringing bioinformatics concepts to school is to equip teachers and students with the competencies to be able to utilise the data and resources in a way that reflects current research practices and communicates an accurate picture of how science is performed [8], [9]. Bioinformatics is especially well-suited to fulfill the requirements of an educational instrument; it is applicable to many aspects of biology curricula, and it supplies a platform for doing research in the classroom, empowering students to access and use real scientific data without the need of a fully equipped wet lab [10]. Exploring web-based bioinformatics resources also builds upon and extends the digital literacy of teachers and their students, and decreases the fear of contact with real scientific resources such as databases and analysis tools. The bioinformatics LearningLABs introduce teachers to the core concepts of computational biology and combine the expertise in educational outreach from ELLS and in bioinformatics from the EMBL-EBI. Furthermore, the LearningLABs provide the opportunity to hear about the latest developments in research, learn about using biological data, encounter life at a research institute, and share experiences with other teachers from around Europe. A central aspect of the courses is to show teachers the (often unexpected) links between curricular topics and cutting-edge research. It is these connections that have the potential to bring science to life and enthuse teachers so they can inspire their students to develop an interest and appreciation for science as something relevant to everyday life. Participation in such courses should be seen as an integral part of teachers' continuous professional development to refresh their content knowledge and further develop their skills in newly emerging and fast-growing areas of science [11].

The development cycle for a bioinformatics LearningLAB builds upon a bioinformatics foundation that aims to communicate the essentials of biological data and resources. This core component is then supplemented by identifying contemporary research topics and by selecting potential activities involving bioinformatics resources that offer appropriate scope for exploration and use by a nonexpert audience (see Text S1 sections “How we design and run bioinformatics courses for teachers” and “An overview of typical types of activities included in a bioinformatics LearningLAB” for descriptions of course composition and components). In addition to EMBL and EMBL-EBI developed activities, we invite external collaborators to contribute their activities where these are aligned with the course focus. Past courses have included representatives from the National Centre for Biotechnology Education (NCBE) at the University of Reading and the Wellcome Trust Sanger Institute.

Alignment of the course content with topics relevant to the teachers' classroom work is a prerequisite for the success of the course and the desired long-term implementation of new concepts into their teaching, as found by other efforts to incorporate computational biology into high school biology lessons [12], [13]. However, as there is no universal European school biology curriculum, this can be challenging to achieve. Our solution is to identify key principles that are shared between most school curricula and represent overarching themes valid for modern biology lessons.

## Methods

The first bioinformatics LearningLAB was run in 2010 and followed by two further courses in 2011 and 2012. We based the original course on the successful ELLS LearningLAB model of short, intensive, practical courses, blending hands-on sessions with research updates from EMBL scientists, providing networking opportunities and visits to world-class research facilities. Although sharing some content and following similar formats, each course was individually designed. The curricular connection points focused on overarching themes such as the molecular basis of genetic/infectious diseases, evolutionary relationships, biological sequences, genome organisation, and structure–function relationships.

**Figure 1:**
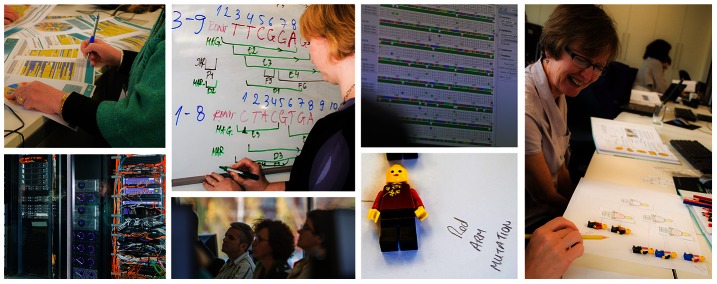
Images from the 2012 LearningLAB on bioinformatics. Top row of images from left to right: course participants taking part in the bioinformatics treasure hunt activity; the “client” role annotates gene information as part of the DAS game; computer-based activity using the Geneious software; learning about phylogenetic trees using LEGO models. Bottom row: visiting the data centre; participants hear a research update; part of a participant's phylogenetic tree diagram built using LEGO minifigures.

From the first course, we have gradually refined our approach to ensure we continue to meet the requirements and expectations of the course participants. Steady improvement of the teaching content is achieved by formative and summative evaluation of the course. We use observation of how activities are received, direct communication with participants during the course, collection of informal teacher-led written feedback (also during the course), and post-course online questionnaires. Suggestions for extensions and adaptations from the teachers are worked into the future versions of the activities. An overview of the course programme and teaching resources presented during the 2012 ELLS LearningLAB “Biology 2.0 – making sense of biological data” can be found online in the course handbook (PDF available for download at www.embl.org/ells/llab261112). Figure 1 shows a selection of images illustrating different elements of the course. Further details on our course design, operational processes, and an overview of the components of the 2010, 2011, and 2012 course programmes can be found in Text S1.

**Figure 2:**
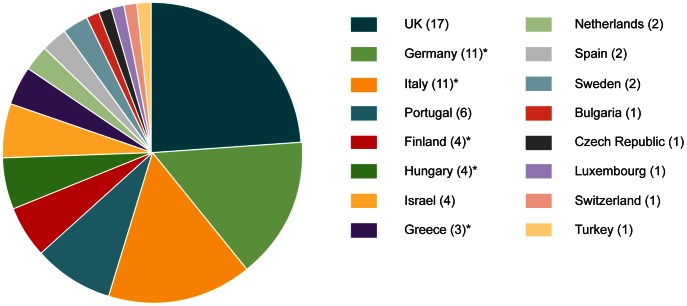
Breakdown of total LearningLAB participants into countries of residence. The diagram shows the countries of residence of all attendees of the 2010–2012 bioinformatics LearningLABs (71 total participants, 16 countries). Labels starred with an asterisk include instances where a participant attended multiple LearningLABs. As each course was different, participants who attended multiple LearningLABs were counted for each course they attended.

When combined, the number of European teachers who have attended the three bioinformatics LearningLABs totals 71, representing 16 countries (Figure 2). To gauge the effectiveness of the LearningLAB training and use of the materials, we surveyed participants of the 2010 and 2011 courses. We received 27 responses from a total of 54 participants. The survey was issued before the 2012 course and therefore the participants of this course are not represented. The rationale for including the cohort of the first two courses was that these teachers would have had sufficient opportunities to incorporate the training materials in their teaching.

## Results/Discussion

The survey showed that the majority of teachers (62%) rated their overall LearningLAB experience as excellent even 12–30 months after the course. Our evaluation revealed that the major impacts on the participants' teaching are in line with our training goals (Table 1 and Figure S1—showing responses to the question “What impact did the course have on your teaching?”). Seventy-three percent of respondents said that the LearningLAB increased their understanding of bioinformatics in general and their knowledge of how to access biological data. The same number of participants selected that the course gave them new activities to try in class. Participants also found it valuable that the course gave them new ideas for activities to use with their students (58%) and increased their awareness of how they could use computer-based resources in their teaching (54%). The survey confirmed our aim of increasing the confidence of teachers when talking to their students about new technologies (50%).

**Table 1:** Summary of LearningLAB training goals and expected impact on teachers and students.

Training goals	Outcomes for teachers	Outcomes for students
1. Extend subject knowledge on biological data and bioinformatics	1. Being able to discuss new techniques and methods with students	1. Greater appreciation and understanding of contemporary research methods
2. Learn how to access and use original biological data	2. Knowing how to convey this knowledge	2. Access to real-life biological data
3. Contact with researchers	3. Information received from a trusted and authentic source	3. Earlier exposure to contemporary research methods and findings
4. Sharing experiences and creating connections with peers from around Europe	4. Widening networks and becoming part of the ELLS teacher network	4. Students benefit from collaborative outcomes
5. Training in a variety of resources and activities to incorporate into lessons	5. Teachers are equipped to adapt and further develop bioinformatics teaching resources	5. New encounters with biology
6. Teachers equipped to disseminate course materials	6. Teachers become the resident expert	6. Wider dissemination to students
7. First hand experience of a research environment	7. Stimulates enthusiasm for new research	7. Experience shared with students to shape their perceptions of research

When asked about which parts of the course the participants found most useful, hands-on activities were rated most highly (76%). This was followed by opportunities to extend teachers' subject knowledge (62%). Other aspects that were ranked as very useful were gaining an introduction to bioinformatics (54%) and learning to use biological data resources (52%). Discussion with teacher colleagues seemed to be an asset (48%), but this was rated below parts of the course related to content and enhancing skills.

We next asked if the participants had used the presented activities in their lessons, how the new content was received by their students, whether this was in a modified format, and the reasons behind why the activities required modification. Over 80% of the course participants had used a LearningLAB resource with their students either once or twice (54%) or frequently (31%). From participants who had used the LearningLAB materials, all respondents reported that the session had been positively received by their students, commenting that the bioinformatics activities allowed their students to experience science in a new way and to connect with current research through the link with available biological data. The majority of participants in our courses also claimed to share the LearningLAB materials with their immediate work colleagues at school (62%) and with local (39%) and national (23%) teacher networks.

More participants reported needing to modify the activities slightly before delivery (71%) compared with delivering the activities unchanged (48%). These response options were not mutually exclusive, so teachers may have used some activities unchanged while wanting to modify others and hence selected both options. Nevertheless, they represent a trend in how the teachers have handled LearningLAB materials. When questioned on the reasons for modifying activities, the predominant reason was that the teachers needed to adjust the biological context or story, for example changing the molecule to investigate (61%). In our opinion, this reflects the diverse topics on curricula in different European countries and most probably the specific preferences and previous experience of individual teachers. In addition, the teachers considered simplifying (56%) and shortening (39%) the teaching activities as important, whereas they seldom (<6%) deviate from the databases and analysis tools we introduce as part of the LearningLAB's training.

More generally, when we asked the teachers about the obstacles they encountered in delivering bioinformatics activities in the classroom, they rated the time investment needed to adapt the activities for use in their teaching most highly (71%). Secondly, they reported that access to computers (59%) is an issue, and 29% said that the need to translate the materials into their teaching language is an obstacle they have to overcome.

Due to the requirement of working with online interfaces and new software programmes, implementing bioinformatics-based activities in the classroom can be hindered by technological barriers [14]. For teachers and students to work with online resources, they require a robust internet connection that very often cannot be guaranteed. Similar problems are encountered through schools' internal regulations on IT administration. Alteration of firewall permissions, choice of web browsers, and installation of specific browser plugins and dedicated analysis software are some of the most common issues facing teachers who want to incorporate bioinformatics resources into their biology lessons. Frequently, teachers rely on a dedicated colleague to help them solve these issues. It is however reassuring that teachers involved in our courses reported that sometimes (54%) or even always (23%) they receive assistance in arranging computer-based sessions.

## Evolving the Bioinformatics LearningLAB Model

Although the bioinformatics LearningLABs have been very positively evaluated by the participants, we have continued to evolve the model by building on our evaluation results to further meet the requirements of the teaching audience. We identified three main opportunities to expand our interaction with the course participants: provision of post-course support, a dedicated course website, and promoting direct access to the resource experts and scientists.

We are aware that teacher training courses are very often one-off encounters. The time spent on the actual course is very limited and the course can cover many concepts. Even if the enthusiasm for directly applying the newly gained knowledge is immense after the course, it is important to help the teachers implement the bioinformatics activities in the classroom. Due to the nature of our international courses and the very dispersed audience, from 2011 onward we have organised follow-up online events to bring course participants back together. The course participants are invited to attend a virtual meeting held approximately six months after the course, which is run using a web conferencing platform. These online events provide a forum for the participants to exchange experiences of using the materials in the classroom, to share ideas and teaching resources developed subsequent to the course, and to give feedback to us as the course organisers. It is also a means of keeping the momentum in the delicate and vulnerable transition from the course to the classroom.

**Figure 3:**
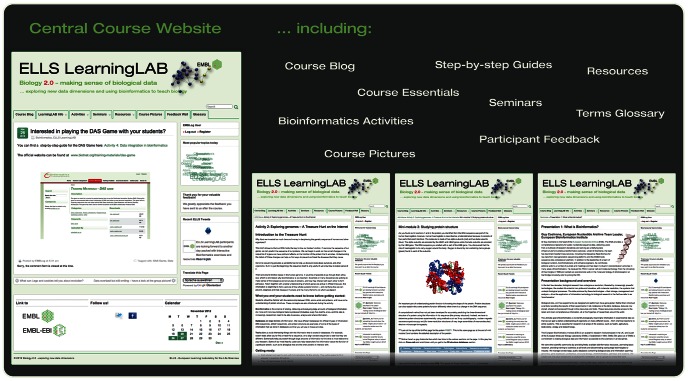
Screenshots of the course website illustrating the main features.

In 2012, we expanded our provision of course materials to include a dedicated course website, which holds all course content and supplementary resources (Figure 3). This provides a much more flexible mechanism for sharing information, responding to requests, and creating a course community. In providing a central repository for course information, it also assists with maintaining the activities, allowing us to update them as required.

More recently we implemented an activity development session as part of the course programme. The objective of this session was to provide the participants with the opportunity to think about how to adapt and amend the course activities to their specific teaching context. EMBL-EBI resource experts provided an informal consultancy service to support the participants in applying their new understanding of bioinformatics to their teaching topics.

As these latter two elements have only been recently incorporated, it is difficult to evaluate their effectiveness; but based upon informal participant feedback, we regard them as offering value to the course programme.

## Conclusions

The general ELLS LearningLAB model offers further opportunities to the participants that extend beyond the actual face-to-face training. Connections developed between course participants are seen by the teachers as a valuable resource in itself. This helps to create strong local, national, or even international relationships, fostering the exchange of adapted or newly developed teaching resources. The maintenance of our relationship with the teachers provides a route for them to further question the researchers and domain knowledge experts after the course.

By applying the LearningLAB model to the field of bioinformatics, we have tested the feasibility of incorporating advanced scientific research methods and biological data at the level of secondary school science education. The benefits resulting from this approach include extending teachers' content knowledge with regards to new developments in biology and the use of biological data, connecting the teachers with a research institute, and enabling them to provide an accurate picture of how modern research is performed. Coupling this experience with tailored bioinformatics training supports them in their role as a conduit, whereby they are able to communicate contemporary scientific knowledge and skills to their students and peer communities. We aim to support the development of ambassadors for bioinformatics. These individuals are well placed to support their fellow teachers in implementing aspects of bioinformatics in their own teaching and also to promote the need for the inclusion of bioinformatics resources in the school curriculum.

Overall, we find that our participants have responded positively to the knowledge and skills offered to them as part of the LearningLAB bioinformatics courses and that, placed in their hands, they have been able to take the next steps to utilise and amend the activities as required by their different teaching approaches, environments, and audiences.

**Considerations for Replicating a Bioinformatics LearningLAB**

In the process of designing and delivering the training courses, responding to feedback, and gradually evolving the model, some key considerations have emerged as being pertinent to achieving a successful learning experience.

1. Course timing and length.

Arrange the course for a convenient time in the academic year, avoiding examination periods and extended holidays. We have found that an intensive two-day course works well, ideally scheduled to allow attendees to travel over a weekend.

2. Ensure an overarching relevance to the teaching curriculum.

Due to the variety of curricula followed by our European participants, we aimed to address core and central themes rather than specific topics.

3. Build upon and develop the existing knowledge areas of the training participants.

We found that in order to support the teachers in connecting new bioinformatics knowledge with their teaching, it was important to demonstrate links to traditional curricular themes while raising awareness of new approaches to teach them.

4. Work within the practical limitations of the classroom.

In order to ensure that it is fit for purpose, course content should be designed with awareness of the constraints teachers face in the classroom. These include accessing computers, having limited software choices, and the rapid evolution of online resources. Some of these constraints can be overcome by considering non-computer-based options such as paper-based alternatives for some activities.

5. Leverage others' pedagogical expertise.

We would recommend involving teachers and other educational experts during the development of teaching activities and in early stages of the course planning. This provides a suitability checkpoint and potentially decreases the subsequent time investment required by teachers before they can incorporate training outputs in their teaching.

6. Provide post-course support.

Depending on the amount of information covered during the limited course time, teachers might benefit from further support during the first steps of implementation of the course materials and knowledge.

7. Evaluate and be open to change.

Formal evaluation provides both the opportunity for participants to reflect on their course experience and essential guidance on how to further develop the course to meet participants' needs.

## Supporting Information

Text S1 The supporting information contains background information on EMBL, ELLS, and the EMBL-EBI; a description of how we design and run the bioinformatics courses for teachers; an overview of the typical types of activities included in a bioinformatics LearningLAB; and supplementary references.
(DOC)

Figure S1 **Responses to the post-course survey question “What impact did the course have on your teaching?”** The radar chart illustrates the main course outcomes as rated by the LearningLAB participants (2010 and 2011).
(TIF)
