# Supplementary material for: Bioinformatics Goes to School—New Avenues for Teaching Contemporary Biology
Source: PLoS Comput Biol. 2013 Jun 13;9(6):e1003089. doi: 10.1371/journal.pcbi.1003089 (PMC3681668; doi:10.1371/journal.pcbi.1003089)
Supplement: Text S1 — The supporting information contains background information on EMBL, ELLS, and the EMBL-EBI; a description of how we design and run the bioinformatics courses for teachers; an overview of the typical types of activities included in a bioinformatics LearningLAB; and supplementary references. (DOC) [file pcbi.1003089.s001.doc]

**Supporting Information**

**Background to EMBL, ELLS and the EMBL-EBI**

The European Molecular Biology Laboratory (EMBL) is Europe’s flagship laboratory, performing basic research in molecular biology in order to understand the fundamental processes of life. The Laboratory’s remit also includes pioneering new research techniques, fostering scientific excellence and providing advanced training. In 2003 EMBL launched the European Learning Laboratory for the Life Sciences (ELLS) to address the growing demand of secondary school science teachers for continuing professional development. ELLS was therefore created to enrich the professional context of teachers, enabling them and their students to experience a research environment first hand. EMBL is based in Heidelberg, Germany, with centres of specialised focus established around Europe: structural biology in Grenoble and Hamburg providing research facilities for European scientists, mouse biology research in Monterotondo and bioinformatics at the European Bioinformatics Institute (EMBL-EBI) in Hinxton, UK. The EMBL-EBI was established in 1994 in response to increasing amounts of biological data being produced by researchers and the need to create accessible data repositories. Today, the EMBL-EBI’s web page receives over 7 million unique hits a day and the institute provides access to biological data resources for millions of users around the world.

**How we design and run bioinformatics courses for teachers**

The course programmes consist of a mix of activity styles, combining hands-on interactive activities, computer-based practical exercises, scientific presentations from research scientists, and discussion of the impact of science on wider societal issues. We draw on a mix of tried and tested materials, refine previously used activities based on teacher feedback and develop new ones as required. The teaching materials are enriched by integrating original scientific resources, such as biological databases, analysis tools and freely available biological data.

Due to its popular training programme, the EMBL-EBI has already accumulated valuable experience in providing resource training to a wide range of audiences [S1]. We incorporated hallmarks of this training experience into the bioinformatics LearningLABs, such as small group sizes and close contact between trainers and trainees in a dedicated training environment. The EMBL-EBI’s training programme has also provided a pool of expert trainers familiar with the institute’s resources and research topics.

During the online application process for the course, we request details from the applicants that allow us to assess their motivations for wanting to attend. These include expectations on how the course will enhance their teaching, information on prior experience in bioinformatics and also their role in local or national teaching networks. We view this latter criterion as highly important in maximising the reach of course materials and in encouraging peer-to-peer sharing of the resources. Generally, the course participants teach biology as a main subject but often are responsible for teaching other sciences too.

Participants are provided with the course materials typically in hard copy to use during the course for reference and also as electronic documents for subsequent use with their students.

**An overview of typical types of activities included in a bioinformatics LearningLAB**

The following activities formed part of the programmes of the 2010, 2011 and 2012 LearningLABs. Different activities from the list were included in different LearningLABs.

Activities using online biological data:

- The evolution of mammoths, exploring the evolutionary relationship between woolly mammoths, Asian and African elephants (from DNA to Darwin, student and teacher guides available at http://www.dnadarwin.org/casestudies/10/)
- The evolution of colour vision (from DNA to Darwin, student and teacher guides available at http://www.dnadarwin.org/casestudies/12/)
- Exploring genomes - a treasure hunt on the internet: a set of modules where the user is guided from resource to resource, starting with a mystery DNA sequence
- Introduction to protein and sequence evolution: sequence alignment and three dimensional protein structures
- Browsing genomes: comparing genomes, viewing sequence and genetic variation using the Ensembl genome browser
- Genetic variation and cancer: looking for mutations in an oncogene using the COSMIC database (http://www.sanger.ac.uk/genetics/CGP/cosmic)
- Protein–protein interactions and understanding how these are described in databases
- Biological pathways

Non-computer based activities:

- A light introduction to phylogenetic trees: creating evolutionary stories to explain the relationship between biscuits of different appearances and flavour (from DNA to Darwin, student and teacher guides available at

<http://www.dnadarwin.org/casestudies/intro>). As an alternative to biscuits, LEGO® minifigures can be used instead (not covered in the online DNA to Darwin materials).

- Interactive game on the distributed annotation system (DAS): learning the basics of data integration and what goes on once a data query is submitted [S2], [S3].
- Malaria challenge: Funding decisions - stepping into the role of research funders and making decisions on which malaria intervention projects to support (available at http://www.yourgenome.org/teachers/fundingdecisions.shtml)
- Genome Generation: a card-based discussion activity that helps students to discuss current issues in genetics and genomics.

Presentations: getting to grips with bioinformatics and current research

- Introduction to bioinformatics, databases and resources
- Understanding human genetic variation
- Genomic research and malaria
- The genetics of cancer

Tours of next-generation sequencing facilities and a data centre.

**Supplementary references**

S1. Via A, De Las Rivas J, Attwood TK, Landsman D, Brazas MD, et al. (2011) Ten Simple Rules for Developing a Short Bioinformatics Training Course. PLoS Comput Biol 7(10): e1002245. doi:10.1371/journal.pcbi.1002245. Available: http://www.ploscompbiol.org/article/info%3Adoi%2F10.1371%2Fjournal.pcbi.1002245. Accessed 1 February 2013.

S2. Schneider MV, Jimenez RC (2012) Teaching the Fundamentals of Biological Data Integration Using Classroom Games. PLoS Comput Biol 8(12): e1002789. doi:10.1371/journal.pcbi.1002789. Available:

http://www.ploscompbiol.org/article/info%3Adoi%2F10.1371%2Fjournal.pcbi.1002789. Accessed 1 February 2013.

S3. The DAS game resources and instructions are available from: http://www.biotnet.org/training-materials/das-game.
