# Supplementary figures and images for: Bioinformatics Goes to School—New Avenues for Teaching Contemporary Biology
Source: PLoS Comput Biol. 2013 Jun 13;9(6):e1003089. doi: 10.1371/journal.pcbi.1003089 (PMC3681668; doi:10.1371/journal.pcbi.1003089)

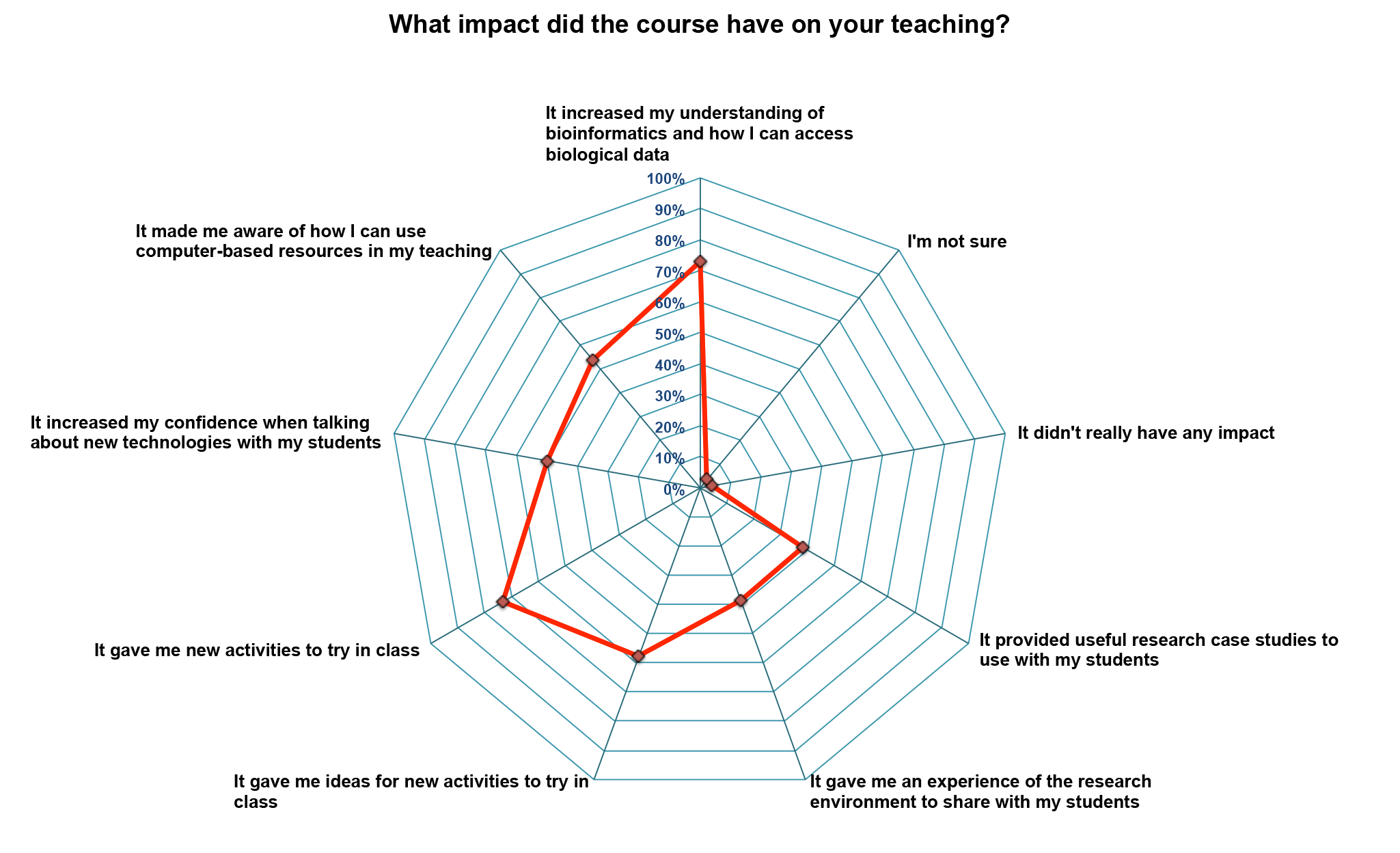

Supplement: Figure S1 — Responses to the post-course survey question “What impact did the course have on your teaching?” The radar chart illustrates the main course outcomes as rated by the LearningLAB participants (2010 and 2011). (TIF) [file pcbi.1003089.s002.tif]
